# Tartrate-resistant acid phosphatase (TRAP) co-localizes with receptor activator of NF-KB ligand (RANKL) and osteoprotegerin (OPG) in lysosomal-associated membrane protein 1 (LAMP1)-positive vesicles in rat osteoblasts and osteocytes

**DOI:** 10.1007/s00418-014-1272-4

**Published:** 2014-09-09

**Authors:** L. B. Solberg, E. Stang, S.-H. Brorson, G. Andersson, F. P. Reinholt

**Affiliations:** 1 Department of Pathology, The Core Facility for Advanced Electron Microscopy, Oslo University Hospital, Rikshospitalet, P.O. Box 4950, Nydalen, 0424 Oslo, Norway; 2 Division of Pathology, F46, Department of Laboratory Medicine, Karolinska Institutet, SE-141 86 Huddinge, Stockholm, Sweden; 3 Department of Pathology, University of Oslo, Oslo University Hospital, Rikshospitalet, P.O. Box 4950, Nydalen, 0424 Oslo, Norway

**Keywords:** TRAP, RANKL, OPG, LAMP1, Osteoblast, Osteocyte

## Abstract

Tartrate-resistant acid phosphatase (TRAP) is well known as an osteoclast marker; however, a recent study from our group demonstrated enhanced number of TRAP + osteocytes as well as enhanced levels of TRAP located to intracellular vesicles in osteoblasts and osteocytes in experimental osteoporosis in rats. Such vesicles were especially abundant in osteoblasts and osteocytes in cancellous bone as well as close to bone surface and intracortical remodeling sites. To further investigate TRAP in osteoblasts and osteocytes, long bones from young, growing rats were examined. Immunofluorescence confocal microscopy displayed co-localization of TRAP with receptor activator of NF-KB ligand (RANKL) and osteoprotegerin (OPG) in hypertrophic chondrocytes and diaphyseal osteocytes with Pearson’s correlation coefficient ≥0.8. Transmission electron microscopy showed co-localization of TRAP and RANKL in lysosomal-associated membrane protein 1 (LAMP1) + vesicles in osteoblasts and osteocytes supporting the results obtained by confocal microscopy. Recent in vitro data have demonstrated OPG as a traffic regulator for RANKL to LAMP1 + secretory lysosomes in osteoblasts and osteocytes, which seem to serve as temporary storage compartments for RANKL. Our in situ observations indicate that TRAP is located to RANKL-/OPG-positive secretory lysosomes in osteoblasts and osteocytes, which may have implications for osteocyte regulation of osteoclastogenesis.

**Electronic supplementary material:**

The online version of this article (doi:10.1007/s00418-014-1272-4) contains supplementary material, which is available to authorized users.

## Introduction

Tartrate-resistant acid phosphatase (TRAP) (ACP5, EC 3.1.3.2) belongs to a group of metalloenzymes, which catalyzes hydrolysis of phosphate esters and anhydrides under acidic conditions. TRAP is synthesized as a relatively inactive pro-enzyme; monomeric TRAP (mTRAP)/loop-TRAP/serum TRAP 5a, but proteolytic processing of the 5a isoform to generate the “two subunit” cleaved TRAP 5b isoform by, e.g., members of the cathepsin family or other proteinases, increases the catalytic activity at least tenfold (Fagerlund et al. 2006; Funhoff et al. 2001; Lang and Andersson 2005; Ljusberg et al. 1999). The isoform TRAP 5b shows different biological abilities in bone, e.g., dephosphorylation of osteopontin (OPN) and integrin-binding sialoprotein (IBSP) (Ek-Rylander and Andersson 2010; Ek-Rylander et al. 1994), dephosphorylation of Man-6-P recognition marker on lysosomal proteins (Bresciani and Von Figura 1996; Sun et al. 2008) and generation of reactive oxygen species for bone matrix degradation (Halleen et al. 1999, 2003; Vaaraniemi et al. 2004). On the other hand, the monomeric TRAP isoform 5a exhibits growth factor-like properties for mesenchymal cells, e.g., pre-adipocytes (Lang et al. 2008; Patlaka et al. 2014) and pre-osteoblasts (Gradin et al. 2012). TRAP gene expression and enzyme activity seems to be most abundant in bone tissue, and young rats express more TRAP mRNA and TRAP enzyme activity than adults (Ek-Rylander et al. 1991; Lang and Andersson 2005). Halleen and co-workers have shown that the serum activity of TRAP 5b is significantly elevated in patients with osteoporosis and negatively correlated with bone mineral density (BMD) (Halleen et al. 2002). Studies on mice yielded similar results: Absence of TRAP leads to disturbed endochondral ossification and a mild osteopetrotic phenotype (Hayman et al. 1996; Suter et al. 2001), while overexpression of TRAP results in enhanced bone turnover and a mild osteoporotic phenotype (Angel et al. 2000). TRAP 5b is mainly found in osteoclasts and has been advocated as a serum marker for osteoclast number. However, also other bone cells express TRAP in vivo; hypertrophic chondrocytes in the rat epiphysis (Hessle et al. 2013), osteoblasts in the metaphysis and in the endosteal and periosteal compartments of diaphyseal rat bone (Bianco et al. 1988; Bonucci et al. 2001; Gradin et al. 2012; Mocetti et al. 2000; Yamamoto and Nagai 1998) as well as osteocytes in cancellous and cortical rat bone near bone surface or bone resorption sites (Bianco et al. 1988; Nakano et al. 2004). The function of TRAP in these cells has been debated and recently increased levels of TRAP gene expression and enzyme activity were demonstrated in osteocytes with active osteocytic osteolysis (Kogawa et al. 2013; Qing et al. 2012). However, the direct contribution of TRAP to osteocytic osteolysis was not investigated in these studies.

The intracellular distribution of TRAP in hypertrophic chondrocytes, osteoblasts and osteocytes is only sparsely described in the literature; however, TRAP enzyme activity has been reported in vesicular structures in osteoblasts (Bonucci et al. 2001; Yamamoto and Nagai 1998) and Reinholt and co-workers demonstrated TRAP + vesicles in osteoblast-like cells with immunogold technique (Reinholt et al. 1990). Our recent TEM studies on osteoblasts and osteocytes revealed TRAP in intracellular electron-dense vesicles with similar features in the two cell types. In addition, there were more TRAP in osteoblasts and osteocytes in rats with experimental osteoporosis, however, without any sign of osteocytic osteolysis. Moreover, TRAP + osteocytes were found in close relation to bone remodeling surfaces and bone surfaces (Solberg et al. 2014). This may indicate a role for TRAP in osteocyte-regulated bone remodeling. To further investigate the role of TRAP in osteoblasts and osteocytes, we hypothesized that a morphological clarification of the TRAP + vesicles would serve as a starting point for elucidation of the function of TRAP in these cells. We therefore performed the current study on young, growing rats, to facilitate the intracellular immunogold labeling, and observed that the TRAP + vesicles in osteoblasts and osteocytes co-labeled for receptor activator of NF-KB ligand (RANKL) as well as lysosomal-associated membrane protein 1 (LAMP1) in the vesicular membrane. We also demonstrated co-localizations of TRAP with RANKL and osteoprotegerin (OPG) in hypertrophic chondrocytes and diaphyseal osteocytes.

## Materials and methods

### Animals

Guide for the Care and Use of Laboratory Animals (2011) was followed and the study protocol approved by the Norwegian National Animal Research Authority. Three days old, normal female Wistar rats were killed by a guillotine. Their right tibia and femur were immediately dissected free and immersed in phosphate-buffered (0.1 M) 4 % paraformaldehyde (pH 7.40) and 0.1 % glutaraldehyde for immunogold labeling and transmission electron microscopic (TEM) analyses or 4 % formalin for immunofluorescence and confocal microscopy, respectively, and stored at room temperature for 24 h. The formalin-fixed femur specimens were embedded in paraffin before they were cut in 2–3-µm-thick longitudinal sections and mounted on plus glass slides.

For immunogold labeling, thawed cryosections were used to increase the success rate for intracellular labeling; after pre-fixation, the paraformaldehyde/glutaraldehyde-fixed tibia specimens were cut into small samples and infiltrated with 2.3 M sucrose over night at 4 °C either directly or after an initial infiltration with 10 % gelatin for 1 h at 37 °C. Infiltration with gelatin was performed to ease orientation and mounting of the specimens. The samples were subsequently mounted on silver stubs, frozen and stored in liquid nitrogen (LN_2_). Ultrathin cryosections (75 nm) were cut with a cryodiamond knife (Diatome ltd, Biel, Switzerland) in a Leica Ultracut EM UC7 ultramicrotome equipped with a Leica EM FC7 cryounit (Leica Microsystems AG, Wetzlar, Germany). Sections were picked up using 2.3 M sucrose and mounted on formvar-coated copper grids before labeling.

### Antibodies

Goat anti-RANKL (SC-7628) and goat anti-OPG (SC-8468) were purchased from Santa Cruz Biotechnology, Inc. (Dallas, Texas, USA). Species anti-LAMP1 (ab24170) was from Abcam (Cambridge, UK). Rabbit anti-loop-TRAP/mTRAP (toward the loop region of uncleaved 5a isoform), and rabbit anti-m + cTRAP (toward both the uncleaved 5a and cleaved 5b isoforms) were from G. Anderssons laboratory (Zenger et al. 2010). Rabbit anti-TRAP (SB-TR103) (toward total TRAP) was purchased from Immunodiagnostic Systems Ltd. (Tyne & Wear, UK). The two antibodies for TRAP (m + cTRAP from G. Anderssons laboratory and total TRAP from Immunodiagnostic Systems) demonstrated equal distribution of the antibody labeling (data not shown). Single labeling was performed for all the different antibodies in order to control for cross-reactivity in the double labeling. Alexa Fluor-555 conjugated donkey anti-rabbit IgG and Alexa Flour-488 donkey anti-goat IgG as well as DAPI nucleic acid stains were from Molecular Probes (Invitrogen Co., Eugene, OR, USA). Protein A-coated 5, 10 and 15 nm colloidal gold were purchased from G. Posthuma (Utrecht, The Netherlands). When labeling for RANKL incubation with a rabbit, anti-goat IgG (Cappel; ICN Biochemicals Costa Mesa, CA, USA) was used as secondary antibody prior to incubation with colloidal gold.

### Immunofluorescence confocal microscopy

The longitudinal sections of paraffin-embedded femurs were rehydrated through a series of graded alcohols, permeabilized in 1 % Triton X-100 in TBST for 20 min and subsequently blocked with 5 % BSA in TBST for 90 min. Primary antibodies were diluted in 1 % BSA, and the sections incubated over night at 2–8 °C. Co-labeling with rabbit anti-TRAP (diluted 1:3,000) and goat anti-OPG (diluted 1:400) or goat anti-RANKL (diluted 1:400) was performed by mixing the primary antibodies before incubation. Non-specific rabbit and goat IgGs were used as negative controls. After extensive washing, bound primary antibodies were visualized by AlexaFluor555 (anti-rabbit) and AlexaFlour488 (anti-goat) and counterstained with DAPI nucleic acid stain for cell count. Sections were mounted with Dako fluorescent-mounting medium (Dako, Denmark) and sealed by cover slips before images were obtained using a NIKON A1R + confocal laser microscope (Nikon Instruments Inc., Melville, NY, USA), equipped with 60 × (water) and 100 ×  (oil) immersion lenses. The images were further processed by the NIS Elements microscope imaging software (Nikon). Double-labeled images were obtained by sequential scanning for each channel to eliminate the “bleed-through” of the chromophores to ensure reliable quantification of the co-localization. Quantification of Pearson’s correlation coefficient PCC (Zinchuk et al. 2007) above threshold was performed using Coloc2 (ImageJ). Strong positive correlation was considered when PCC ≥0.8. Background subtraction was done by using the built-in rolling ball radius algorithm and set equal in all images. Region of interest (ROI) was selected using lasso tool around each cell subjected to analyses. Hypertrophic chondrocytes in the growth plate region and diaphyseal osteocytes were analyzed for the two different antibody combinations; TRAP/RANKL and TRAP/OPG. Ten cells from each animal and cell type were analyzed and their means calculated. The negative controls demonstrated no staining. Three-dimensional reconstructions of the confocal sectioning images (z-stacks) were performed using the Volume Viewer plugin (ImageJ).

### Immunogold labeling of thawed cryosections

Immunogold labeling of thawed cryosections was performed principally as previously described (Griffiths 1993). In brief, for single labeling, sections were incubated with 1 % BSA in PBS for 30 min at room temperature before incubation with primary antibody dissolved in 1 % BSA for 30 min at room temperature followed by protein A-coated 5, 10 or 15 nm colloidal gold particles for 30 min at room temperature. For double-labeling, sections labeled with the first primary antibody and colloidal gold, as described for single labeling, were incubated with free protein A in 1 % BSA for 30 min at room temperature. Before incubation with the second primary anybody followed by colloidal gold of a different size (for details, see Figure legends), both incubations for 30 min at room temperature. Analyses were performed using conventional TEM (Tecnai 12, FEI Company, Eindhoven, Netherlands).

### Measurements of osteocyte canaliculi diameters

The physical limitation for vesicular transport in the osteocyte canaliculi were investigated by measuring the transverse diameter of the osteocyte canaliculi in cortical and cancellous bone. After pre-fixation, the paraformaldehyde/glutaraldehyde long bone specimens were decalcified in 7 % EDTA with 0.5 % paraformaldehyde before they were cut into small samples (~1 mm^3^) and embedded in conventional epoxy resin. Ultrathin sections (75 nm) were cut and mounted on formvar-coated nickel slot grids and subjected to TEM analyses. The diameter of 10 circular cross-sections of the osteocyte canaliculi were measured for each animal in both cortical and cancellous bone, and the results presented as means with standard deviations (SD). The statistical analyses were performed in PASW Statistics 18 (SPSS Inc., Chicago, IL, USA) using a Student’s *t* test for two variables.

## Results

### Immunofluorescence

#### Co-localization of TRAP with RANKL and OPG in hypertrophic chondrocytes

**Fig. 1:**
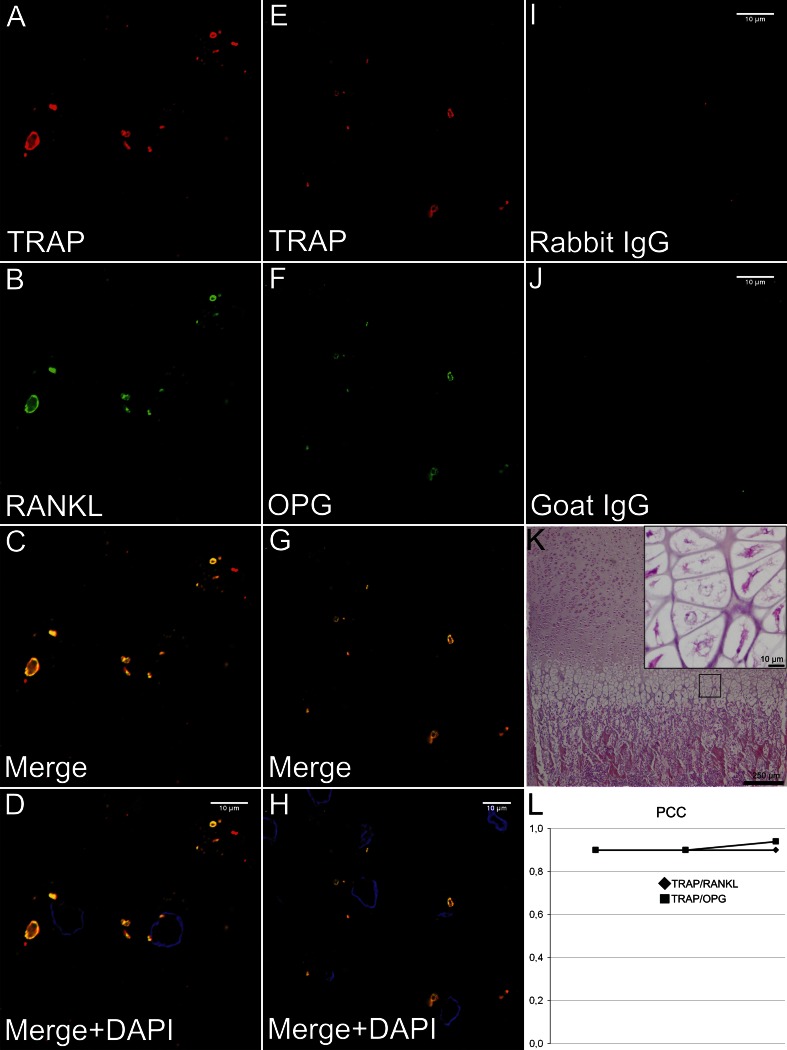
Co-localization of TRAP with RANKL and OPG in hypertrophic chondrocytes. **a**–**d** Immunofluorescence images with hypertrophic chondrocytes labeled for TRAP (m + cTRAP, *red*), RANKL (*green*) and the merge of the channels (*yellow*) as well as the merge with DAPI (*blue*) for cell separation. **e**–**h** Immunofluorescence images with hypertrophic chondrocytes labeled for TRAP (m + cTRAP, *red*), OPG (*green*) and the merge of the channels (*yellow*) as well as the merge with DAPI (*blue*) for cell separation. Both antibody pairs co-localize in what seem to be vesicular structures in the hypertrophic chondrocytes. **i**, **j** Unspecific rabbit IgG and goat IgG served as negative controls for TRAP and RANKL/OPG, respectively, and did not demonstrate any specific labeling. **k** Light microscopic image of conventional HES stained corresponding section demonstrating the tissue architecture in the epiphysis and metaphysis of the distal femur. Light microscopic image of the hypertrophic zone in the growth plate at a higher power is inserted. **l** Pearson’s correlation coefficient (PCC) demonstrated strong co-localization (PCC ≥0.8) for both TRAP/RANKL and TRAP/OPG in the hypertrophic chondrocytes subjected to the co-localization analyses (*n* = 10 in three different animals)

Confocal microscopy revealed an intense staining for TRAP in hypertrophic chondrocytes in the femur epiphyses. Single labeling for RANKL and OPG displayed a similar pattern. Double labeling for TRAP/RANKL and TRAP/OPG demonstrated visual co-localization between the antibodies in the two pairs. Quantitative co-localization analyses for TRAP/RANKL and TRAP/OPG in the hypertrophic chondrocytes in the epiphyseal growth plate confirmed the visual observed pattern with mean PCC = 0.91 for TRAP/RANKL and mean PCC = 0.92 for TRAP/OPG (Fig. 1). Reconstruction of z-stacks demonstrated co-localization for the two antibody pairs also in the third dimension (Online Resource 1).

#### Co-localization of TRAP with RANKL and OPG in osteocytes

**Fig. 2:**
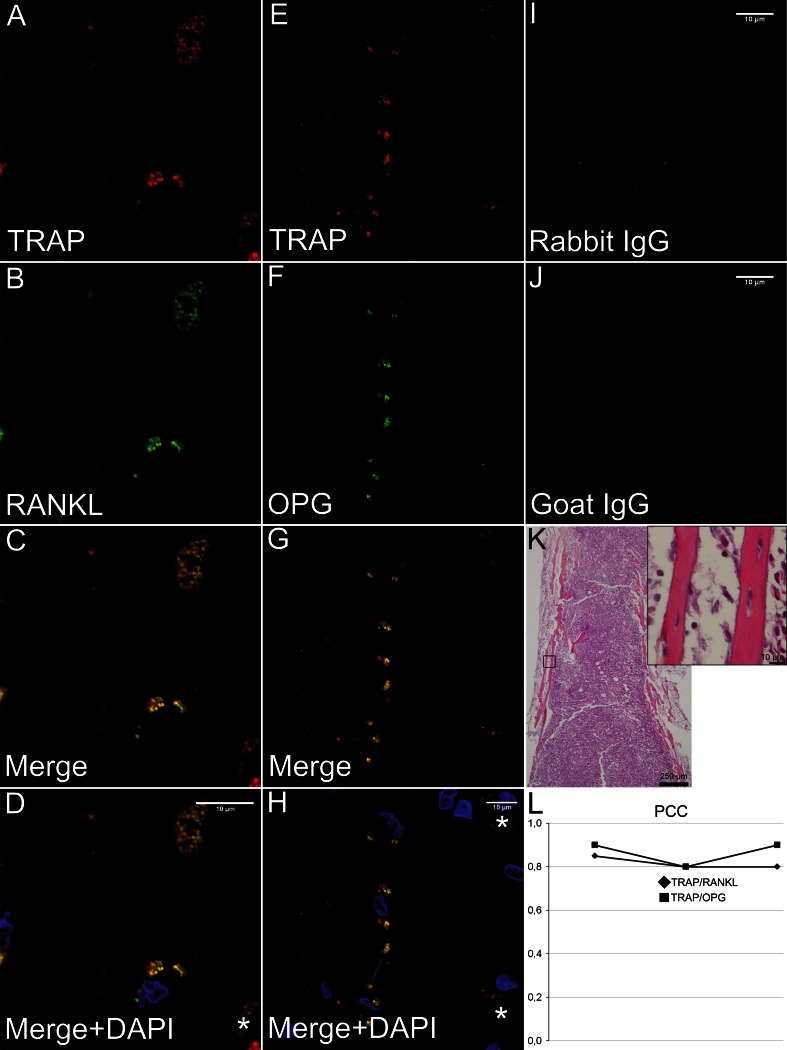
Co-localization of TRAP with RANKL and OPG in diaphyseal osteocytes. **a**–**d** Immunofluorescence images with diaphyseal osteocytes labeled for TRAP (m + cTRAP, *red*), RANKL (*green*) and the merge of the channels (*yellow*) as well as the merge with DAPI (*blue*) for cell separation. **e**–**h** Immunofluorescence images with diaphyseal osteocytes labeled for TRAP (m + cTRAP, *red*), OPG (*green*) and the merge of the channels (*yellow*) as well as the merge with DAPI (*blue*) for cell separation. **i**, **j** Unspecific rabbit IgG and goat IgG served as negative controls for TRAP and RANKL/OPG, respectively, and did not demonstrate any specific labeling. **k** Light microscopic image of conventional HES stained corresponding section demonstrating the tissue architecture of the diaphysis of the femur. Light microscopic image of one of the cortices at a higher power is inserted. **l** Pearson’s correlation coefficient (PCC) demonstrated strong co-localization (PCC ≥0.8) for both TRAP/RANKL and TRAP/OPG in the osteocytes subjected to the co-localization analyses (*n* = 10 in three different animals)

**Fig. 3:**
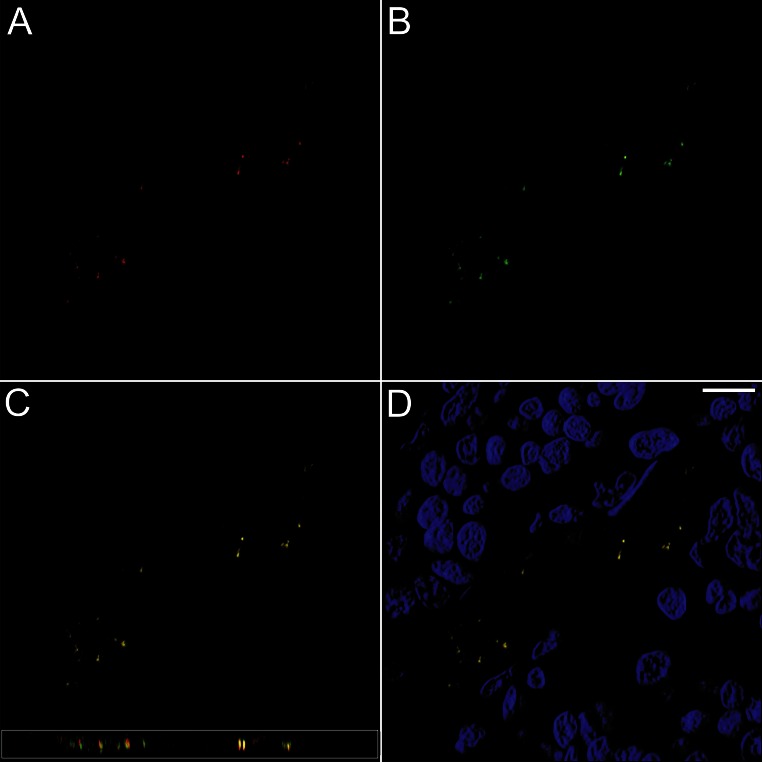
Immunofluorescence images from metaphyseal bone. **a** TRAP (m + cTRAP), *red*. **b** OPG, *green*, with **c** the merger between the channels with co-localization (*yellow*) between the antibodies in what seems to be vesicular structures in osteocyte canaliculi. The image along the zx-axis is inserted. **d** The merger between the channels with DAPI (*blue*) for cell nuclei demonstrates the bony trabecula surrounded by bone marrow. *Scale bar* 10 µm

In the diaphysis, TRAP-positive osteocytes were observed clustered in site-specific areas, and the same pattern was also observed for RANKL- and OPG-positive osteocytes with visual co-localization between TRAP/RANKL and TRAP/OPG. However, also other TRAP-positive cells were observed; these cells were multinucleated and mainly observed at the trabecular surface facing the bone marrow and did not label neither for RANKL nor for OPG and were judged to be osteoclasts. To confirm the observed pattern of co-localization between TRAP/RANKL and TRAP/OPG in osteocytes within the bone tissue, quantitative co-localization analyses were performed on the osteocytes that demonstrated visual co-localization. Strong co-localization was calculated for the two antibody pairs with mean PCC = 0.81 for TRAP/RANKL and mean PCC = 0.88 for TRAP/OPG (Fig. 2). Reconstructions of z-stacks demonstrated co-localization for the two antibody pairs also in the third dimension (Online Resource 2). In addition to the intracellular co-localization in osteocytes, we also observed co-localization of TRAP and OPG in what seemed to be osteocyte canaliculi, indicating intracellular transport of the proteins in the canaliculi (Fig. 3).

### Transmission electron microscopy (TEM)

#### Morphological features of TRAP + vesicles in osteoblasts and osteocytes

**Fig. 4:**
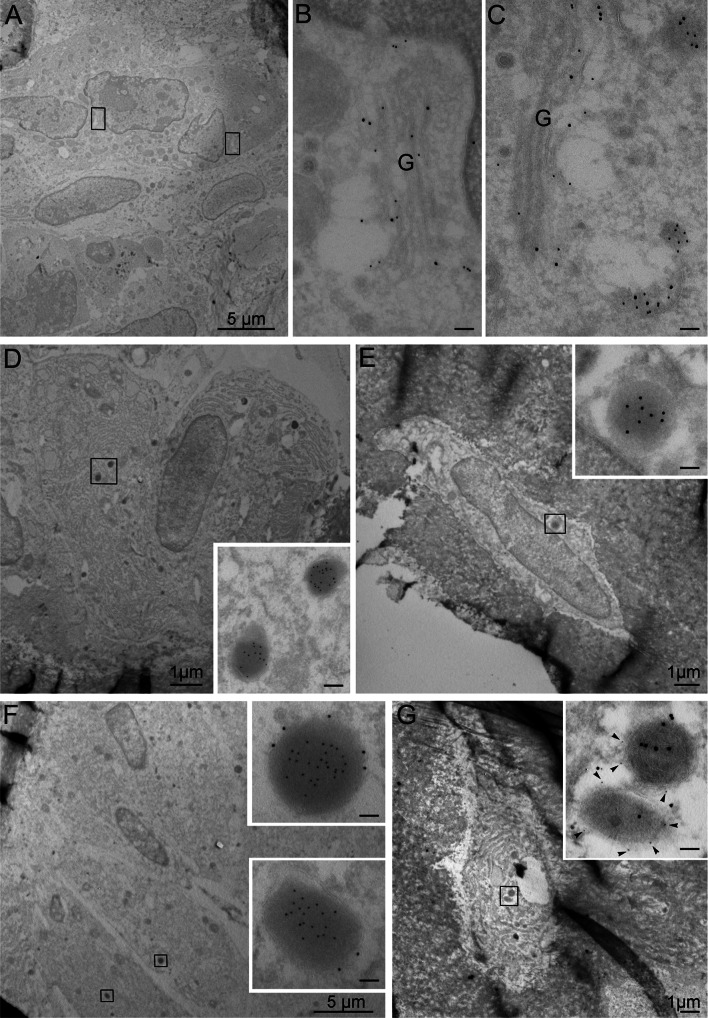
Micrographs from TEM analyses. **a**–**c** Osteoclast in the proximal tibia metaphysis with double labeling for TRAP (total TRAP), 10 nm colloidal gold, and loop-TRAP/mTRAP, 15 nm colloidal gold. **b** and **c** represent the *marked areas* in **a** at a higher magnification. **d** Osteoblasts in the proximal tibia metaphysis labeled for total TRAP, 15 nm colloidal gold. **e** Osteocyte from tibia diaphysis labeled for TRAP (m + cTRAP), 15 nm colloidal gold. **f** Osteoblasts in proximal tibia metaphysis with double labeling for TRAP (total TRAP), 10 nm colloidal gold, and LAMP1, 15 nm colloidal gold. **g** Osteocyte in proximal tibia metaphysis with co-labeling for TRAP (m + cTRAP) and LAMP1, 5 nm colloidal gold, *arrowheads*. *Scale bars* in **b**, **c** and inserts 100 nm,* G* Golgi

TEM analyses showed labeling for TRAP in the Golgi complex in osteoclasts and in electron-dense vesicles in osteoblasts and osteocytes (Fig. 4a–e). The TRAP + vesicles in osteoblasts and osteocytes were 200–500 nm in diameter and did not appear to be restricted to any specific location in the cytoplasm. No fusion between the TRAP + vesicles and the cell membrane was observed. Monomeric TRAP (loop-TRAP/mTRAP) was detected in the Golgi complex in osteoclasts (Fig. 4b, c), but not in this organelle in osteoblasts or osteocytes. LAMP1 was observed in the vesicular membrane of the TRAP + vesicles in osteoblasts and osteocytes (Fig. 4f, g), indicating that the vesicles may represent late endosomes, lysosomes, or secretory lysosomes.

#### Morphological features of RANKL + vesicles in osteoblasts and osteocytes

**Fig. 5:**
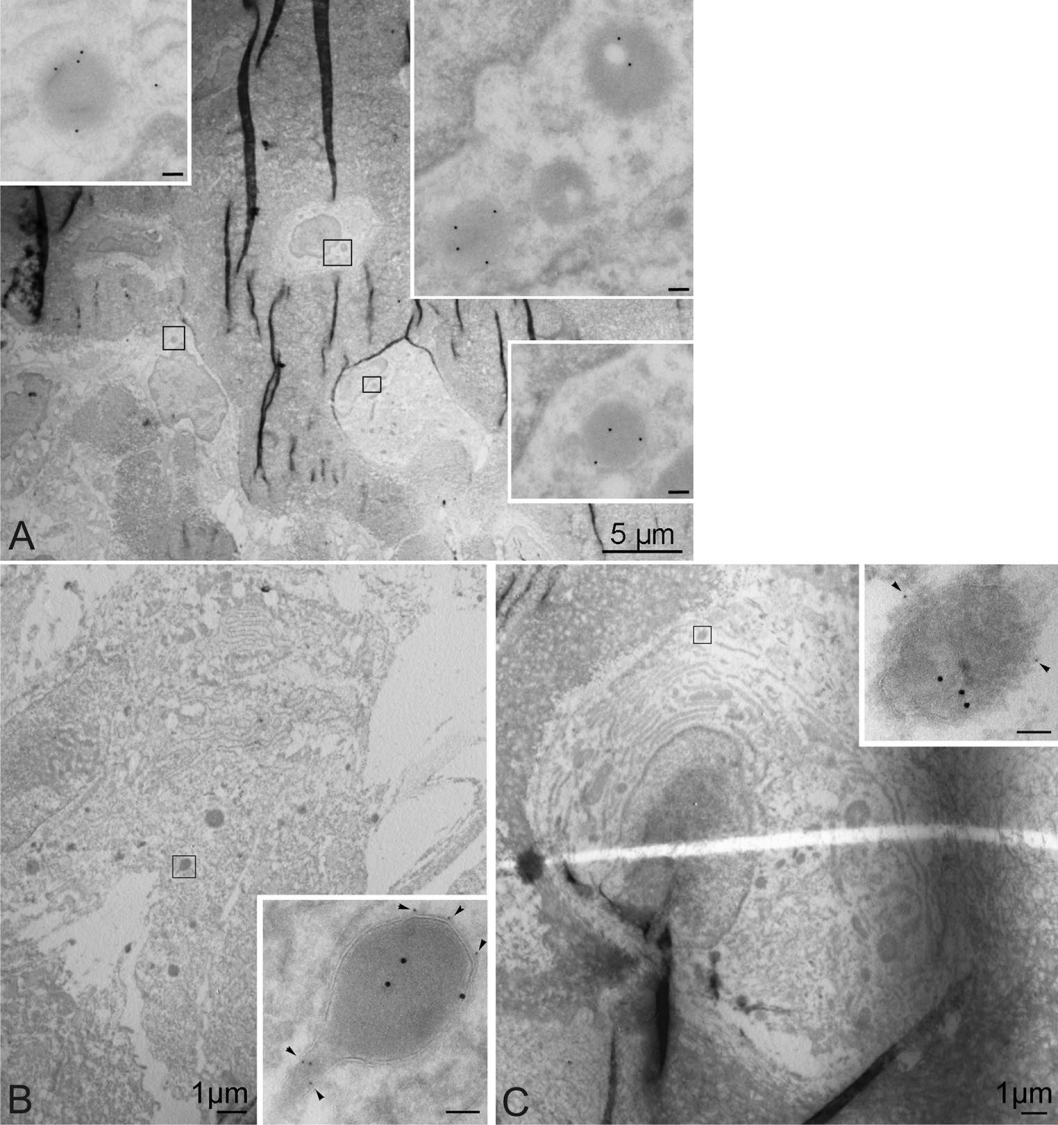
Micrographs from TEM analyses. **a** Tibia diaphysis labeled for RANKL, 15 nm colloidal gold, demonstrates labeling in both osteoblasts and osteocytes (inserted at a higher power). **b** Osteoblast from proximal tibia metaphysis double-labeled for RANKL, 15 nm colloidal gold, and LAMP1, 5 nm colloidal gold, *arrowheads*. **c** Osteocyte from proximal tibia metaphysis with double labeling of RANKL, 15 nm colloidal gold, and LAMP1, 5 nm colloidal gold, *arrowheads*. *Scale bars* in inserts 100 nm

TEM analyses demonstrated RANKL in electron-dense vesicles in osteoblasts and osteocytes (Fig. 5a) similar to those observed positive for TRAP. These vesicles also displayed labeling for LAMP1 in their membranes (Fig. 5b, c).

#### TRAP and RANKL co-localize in intracellular vesicles in osteoblasts and osteocytes

**Fig. 6:**
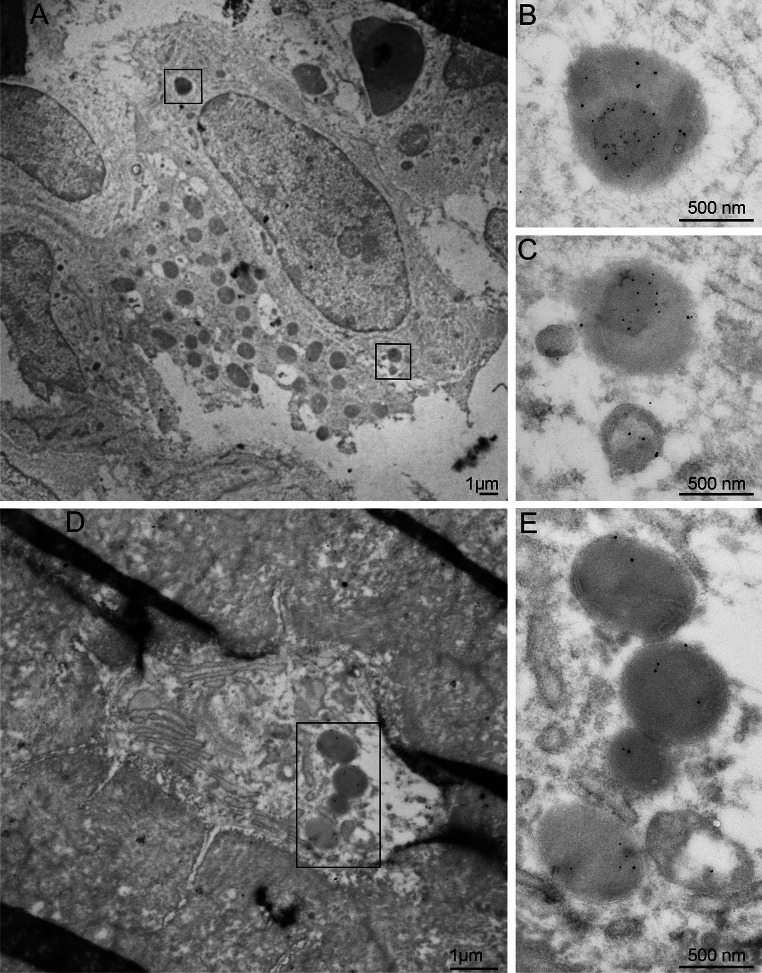
Micrographs from TEM analyses. **a** Osteoblast in proximal tibia metaphysis double-labeled for TRAP (m + cTRAP), 10 nm colloidal gold, and RANKL, 15 nm colloidal gold. **b** and **c** represent the *marked areas* in **a** at a higher power. **d** Osteocyte in tibia diaphysis double-labeled for TRAP (m + cTRAP), 10 nm colloidal gold, and RANKL, 15 nm colloidal gold. **e** represents the *marked area* in **d** at a higher magnification

As the observed TRAP + and RANKL + vesicles shared similar morphological features as well as presented LAMP1 in their membranes, co-labeling for TRAP with RANKL was performed using ultrastructural immunogold labeling. This demonstrated co-localization of TRAP and RANKL in the vesicles in both osteoblasts and osteocytes supporting the results from the confocal laser microscopy (Fig. 6a–e).

### The diameter of the osteocyte canaliculi

The transverse diameter of the osteocyte canaliculi in cortical and cancellous bone demonstrated no significant difference between the canaliculi diameter in the two bone types with means of 219 ± 45 and 236 ± 39 nm in cortical and cancellous bone, respectively. The results showed little variance with SDs of 20 and 17 %. The absolute diameter varied between 147 and 397 nm in cortical bone, and between 159 and 324 nm in cancellous bone.

## Discussion

This study shows for the first time co-localization of TRAP with RANKL in vesicular structures in osteoblasts, osteocytes and hypertrophic chondrocytes in vivo, as well as co-localization of TRAP with OPG in hypertrophic chondrocytes and osteocytes. In addition, LAMP1 was demonstrated in the membranes of the TRAP + and RANKL + compartments.

RANKL is known as one of the key regulators of the osteoclastogenesis initiating bone remodeling, while OPG is known as the RANKL decoy receptor that inhibits the activation of osteoclasts by RANKL and regulates bone resorption. RANKL is produced and secreted from various types of cells, among them are chondrocytes, osteoblasts and osteocytes (Ikeda et al. 2001; Kartsogiannis et al. 1999; Kishimoto et al. 2006; Silvestrini et al. 2005). Recent studies have shown that osteocytes is the main source of RANKL in remodeling cancellous bone in adult mice (Nakashima et al. 2011; Xiong et al. 2011) as well as promoting the bone loss caused by secondary hyperparathyroidism (Xiong et al. 2014). However, the exact intracellular mechanisms involved in the regulation and secretion of RANKL are not fully understood, although the group of Suzuki has performed extensive research on this topic in osteoblasts and osteocytes in vitro (Aoki et al. 2010; Honma et al. 2013; Kariya et al. 2009, 2011). They have demonstrated two pathways for the transport of RANKL to the cell surface in both osteoblasts and osteocytes; one minor route transporting RANKL directly via the Golgi complex to the cell surface; and one major route requiring Vps33a (Kariya et al. 2009) in addition to OPG binding to RANKL before the complex is sorted to LAMP1 + secretory lysosomes (Aoki et al. 2010). The sorting of RANKL to secretory lysosomes in the major route seems to be the crucial pathway in the regulation of the osteoclastogenesis, as a defect in the traffic regulatory activity of OPG increases the osteoclastogenic ability in vitro despite increased numbers of OPG decoy receptors (Aoki et al. 2010). The release of RANKL through the fusion of secretory lysosomes with the plasma membrane is suggested to be regulated by the binding of RANK at the surface of osteoclast precursors to small amounts of RANKL presented at the osteoblast or osteocyte membrane from the minor pathway. This will in turn activate Rab27a/b and lead to docking and fusion of the secretory lysosomes with the plasma membrane and release of RANKL (Honma et al. 2013; Kariya et al. 2011). Interestingly, also TRAP is sorted into the secretory pathway and lysosomal pathway in MDA MB-231 breast cancer cells (Zenger et al. 2010).

LAMP1 is known to be present in membranes of late endosomes and lysosomal structures promoting their acidic interior and protecting the membranes from auto-digestion (Fukuda 1991). Generally, late endosomes and lysosomes are considered to be degradative organelles where lysosomes represent the end-stage of the endocytotic route degrading endocytosed material. Different subclasses of these organelles, however, do exist and both late endosomes and lysosomes can function as secretory organelles. Specialized multivesicular late endosomes can fuse with the plasma membrane and cause the release of exosomes (Raposo and Stoorvogel 2013), and secretory lysosomes can secrete their contents in response to external stimuli (Holt et al. 2006). Secretory lysosomes and “ordinary” lysosomes share distinct features such as the presence of lysosomal-associated membrane proteins and an acidic luminal pH for the optimal function of the acid hydrolases stored in their lumen. Secretory lysosomes are, however, specialized as they, in addition to having a degradative function, serve as organelles for storage of newly synthesized secretory proteins and possess an ability to fuse with the plasma membrane (Blott and Griffiths 2002). The presence of LAMP1 in the TRAP + vesicle membrane does not alone allow a direct assignment of the nature of the vesicles. However, we suggest that the TRAP + vesicles co-labeling for both RANKL and LAMP1 in osteoblasts and osteocytes in vivo are similar to the secretory lysosomes described as storage compartments for RANKL by the group of Suzuki (Aoki et al. 2010). In addition, since OPG has been described to regulate traffic of RANKL from the Golgi complex to the secretory lysosomes (Aoki et al. 2010), this notion is further supported by the immunofluorescence analyses showing strong co-localization for both TRAP/RANKL and TRAP/OPG in diaphyseal osteocytes. The data may imply a role for TRAP in the secretory lysosomes or an extracellular effect of TRAP if TRAP is released together with RANKL from the secretory lysosomes.

If the vesicles are secretory lysosomes secreting their content at the cell surface in order to communicate with other cells, e.g., in the bone marrow, they would be expected to be small enough to travel along the osteocyte canaliculi. In addition, a directional transport mechanism should be present. You et al. (2004) have estimated the canalicular diameter in various species from other publications as well as measured osteocyte canaliculi in long bone diaphyses from 15 weeks old mice and got variable results; the estimated diameter of the osteocyte canaliculi varied from 700 nm in human tibia to 100 and 125 nm in human compact bone and new born rabbits, respectively. In their own results from 15-week old mice, the canaliculi diameter varied between 80 and 710 nm. The large variation may be due to use of different species, sex as well as to the measurement techniques; to ensure that the diameter are not measured too small or too wide, only profiles with transverse, circular osteocyte canaliculi should be measured. In our material, we found the osteocyte canalicular diameter to vary between 146 and 397 nm in cortical bone and with similar results for cancellous bone and with means of 219 and 236 nm, respectively. The diameter of the observed TRAP-RANKL-LAMP1 + vesicles varied from 200 to 500 nm. As the vesicles have lipid membranes, they are plastic and may also be able to split to fit into the osteocyte canaliculi, which also varies in size. According to the present results, it seems plausible that the vesicles travel along the osteocyte extensions to reach the bone surface or bone remodeling surfaces. One might speculate that the actin skeleton (Baik et al. 2013; Murshid et al. 2007) in the osteocyte extensions could play a role in a directional transport of proteins from the cell body to the bone surface equal to the well-studied directional transport along microtubules and actin filaments in neuronal axons and dendrites (Hirokawa and Takemura 2005). This would make cell–cell communication possible and may provide an opportunity for RANK and membrane-bound RANKL to interact in order to stimulate osteoclastogenesis. However, further investigations on transport mechanisms in osteocytes are needed to clarify some of these questions.

The demonstration of loop-TRAP/mTRAP in the Golgi complex in osteoclasts but not in osteoblasts and osteocytes may be related to a lower synthesis in the latter cells, as a low-grade expression is difficult to detect with the immunogold technique. Furthermore, labeling for TRAP in osteoblasts and osteocytes was restricted to LAMP1 + electron-dense vesicles, while no significant labeling was observed neither along the synthetic pathway (endoplasmatic reticulum and Golgi) nor early in the endocytotic pathway (plasma membrane, early endosomes, multivesicular bodies). This may indicate that the technique is not sensitive enough to detect small amounts of protein along these pathways and indirectly supports the assumption that the observed labeling of TRAP is localized to secretory lysosomes where newly synthesized protein gets stored and thus concentrated to a degree that allows detection. TRAP gene expression in osteoblasts and osteocytes is well documented by RT-PCR (Kogawa et al. 2013; Qing et al. 2012) and in situ hybridization (Gradin et al. 2012; Nakano et al. 2004). Moreover, osteocytes in both cancellous and cortical bone express loop-TRAP/mTRAP (Solberg et al. 2014), and it is therefore not likely that the TRAP + vesicles contain TRAP with another origin than from within the osteoblasts and osteocytes themselves. This notion is also supported by Bonucci et al. (2001).

In addition to the demonstration of TRAP + vesicles in osteoblasts and osteocytes, we observed TRAP in what seems to be vesicular structures in hypertrophic chondrocytes. The presence of TRAP in hypertrophic chondrocytes has been reported by others (Hessle et al. 2013). Moreover, mice lacking TRAP (TRAP –/– mice) present widening of the epiphyseal growth plates due to a widening of the hypertrophic zone with disorganized columns (Blumer et al. 2012; Hayman et al. 1996; Hollberg et al. 2002). This may reflect disturbed chondrocyte maturation and function as well as a defect in the chondroclast/osteoclast resorption, indicating a dual effect of the enzyme. RANKL has previously been demonstrated in hypertrophic chondrocytes (Kishimoto et al. 2006; Silvestrini et al. 2005; Xiong et al. 2011) and also reported to be located to the same cell type and bone level as OPG (Silvestrini et al. 2005). We demonstrate for the first time co-localization of TRAP with RANKL and OPG in hypertrophic chondrocytes in what seems to be vesicular structures. As hypertrophic chondrocytes have been suggested to have a similar function in cartilage as osteocytes in bone by initiating remodeling (Xiong et al. 2011), we suggest the same mechanisms for TRAP in association with RANKL and OPG to be operative in hypertrophic chondrocytes as in osteoblasts and osteocytes.

In conclusion, we propose that the vesicular TRAP we observe in osteoblasts, osteocytes and hypertrophic chondrocytes in relation to RANKL and OPG may either have a role intracellularly in secretory lysosomes where the enzyme carry out its task in loco as an acidic phosphatase, or is secreted in response to external stimuli to perform its function extracellularly.

## Electronic supplementary material

Below is the link to the electronic supplementary material.

Supplementary material 1 (DOCX 13 kb)

Supplementary material 2 (PDF 539 kb)

Supplementary material 3 (PDF 597 kb)
