# Supplementary material for: Tartrate-resistant acid phosphatase (TRAP) co-localizes with receptor activator of NF-KB ligand (RANKL) and osteoprotegerin (OPG) in lysosomal-associated membrane protein 1 (LAMP1)-positive vesicles in rat osteoblasts and osteocytes
Source: Histochem Cell Biol. 2014 Sep 9;143(2):195–207. doi: 10.1007/s00418-014-1272-4 (PMC4298672; doi:10.1007/s00418-014-1272-4)
Supplement: Supplementary file 1 — Supplementary material 1 (DOCX 13 kb) [file 418_2014_1272_MOESM1_ESM.docx]

**FIGURE CAPTIONS FOR ONLINE RESOURCES**

**Online Resource 1** Immunofluorescence images of hypertrophic chondrocytes demonstrating co-localization of TRAP with RANKL and OPG also along the zy-axis. A-E: TRAP (m+cTRAP, red), RANKL (green), the merger between the cannels (yellow) and the merge+DAPI for cell nuclei stain to the right. E represents the marked area in D and shows the co-localization of the antibodies along the zy-axis. F-J: TRAP (m+cTRAP, red), OPG (green), the merger between the cannels (yellow) and the merge+DAPI for cell nuclei stain to the right. J represents the marked area in I and shows the co-localization of the antibodies along the zy-axis. Scale bars 10µm

**Online Resource 2** Immunofluorescence images of diaphyseal osteocytes demonstrating co-localization of TRAP with RANKL and OPG also along the zy-axis. A-E: TRAP (m+cTRAP, red), RANKL (green), the merger between the cannels (yellow) and the merge+DAPI for cell nuclei stain to the right. E represents the marked area in D and shows the co-localization of the antibodies along the zx-axis. F-J: TRAP (m+cTRAP, red), OPG (green), the merger between the cannels (yellow) and the merge+DAPI for cell nuclei stain to the right. J represents the marked area in I and shows the co-localization of the antibodies along the zx-axis. Scale bars 10µm
