# Supplementary figures and images for: Tartrate-resistant acid phosphatase (TRAP) co-localizes with receptor activator of NF-KB ligand (RANKL) and osteoprotegerin (OPG) in lysosomal-associated membrane protein 1 (LAMP1)-positive vesicles in rat osteoblasts and osteocytes
Source: Histochem Cell Biol. 2014 Sep 9;143(2):195–207. doi: 10.1007/s00418-014-1272-4 (PMC4298672; doi:10.1007/s00418-014-1272-4)

A

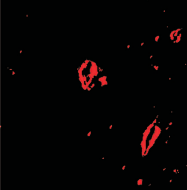

TRAP

B

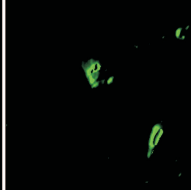

RANKL

C

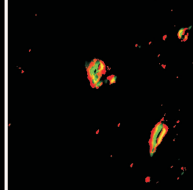

Merge

D

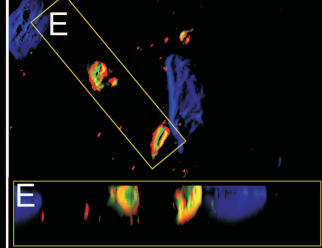

F

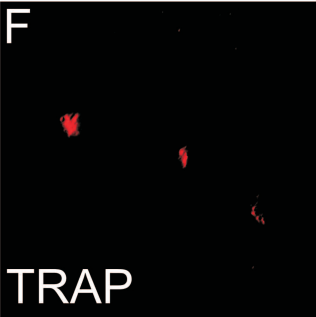

TRAP

G

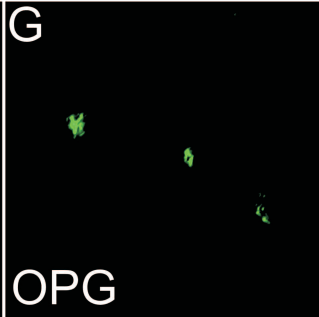

OPG

H

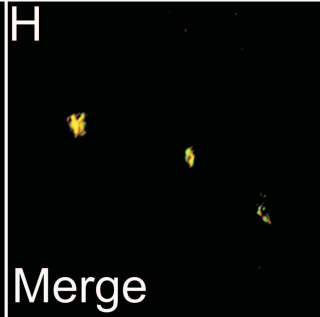

Merge

I

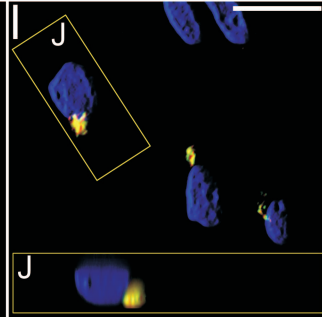

Supplement: Supplementary file 2 — Supplementary material 2 (PDF 539 kb) [file 418_2014_1272_MOESM2_ESM.pdf]

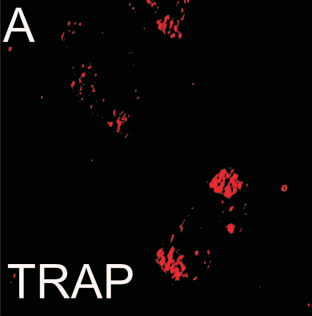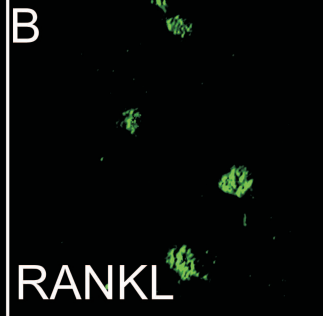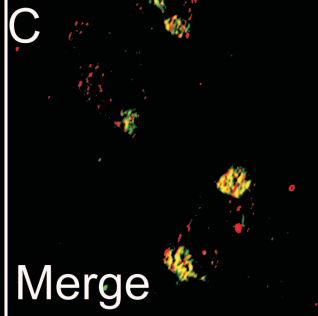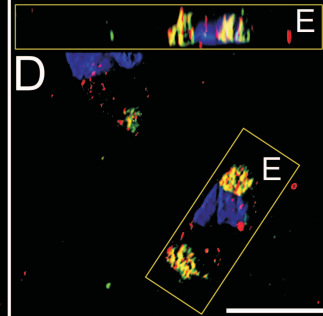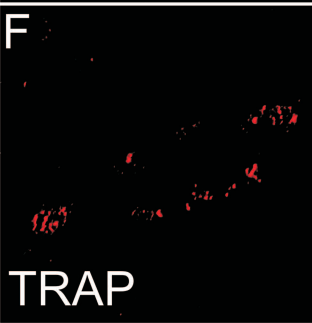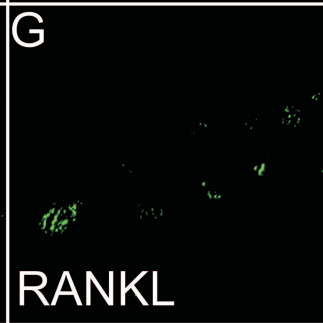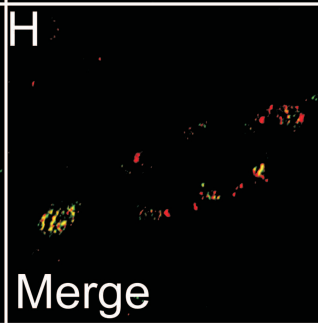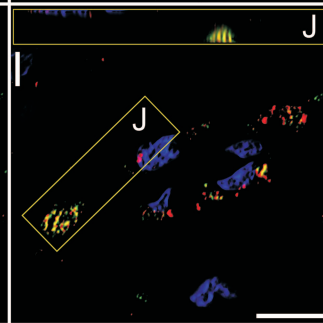

Supplement: Supplementary file 3 — Supplementary material 3 (PDF 597 kb) [file 418_2014_1272_MOESM3_ESM.pdf]
